# Stacking angle-tunable photoluminescence from interlayer exciton states in twisted bilayer graphene

**DOI:** 10.1038/s41467-019-09097-x

**Published:** 2019-03-29

**Authors:** Hiral Patel, Lujie Huang, Cheol-Joo Kim, Jiwoong Park, Matt W. Graham

**Affiliations:** 1 Department of Physics, Oregon State University, Corvallis, OR 97331 USA; 2 Department of Chemistry, Institute of Molecular Engineering, University of Chicago, Chicago, IL 60637 USA; 3 Department of Chemical Engineering, Pohang University of Science and Technology, Pohang 790-784, Korea

## Abstract

Twisted bilayer graphene (*t*BLG) is a metallic material with two degenerate van Hove singularity transitions that can rehybridize to form interlayer exciton states. Here we report photoluminescence (PL) emission from *t*BLG after resonant 2-photon excitation, which tunes with the interlayer stacking angle, *θ*. We spatially image individual *t*BLG domains at room-temperature and show a five-fold resonant PL-enhancement over the background hot-electron emission. Prior theory predicts that interlayer orbitals mix to create 2-photon-accessible strongly-bound (~0.7 eV) exciton and continuum-edge states, which we observe as two spectral peaks in both PL excitation and excited-state absorption spectra. This peak splitting provides independent estimates of the exciton binding energy which scales from 0.5–0.7 eV with *θ* = 7.5° to 16.5°. A predicted vanishing exciton-continuum coupling strength helps explain both the weak resonant PL and the slower 1 ps^−1^ exciton relaxation rate observed. This hybrid metal-exciton behavior electron thermalization and PL emission are tunable with stacking angle for potential enhancements in optoelectronic and fast-photosensing graphene-based applications.

## Abstract (web summary)

Interlayer electronic states in twisted bilayer graphene are characterized by flat-band regions hosting many-body electronic effects. Here, the authors observe two-photon photoluminescence excitation and excited-state absorption spectra on graphene containing a variety of twist angles to access the dark exciton transitions and estimate the exciton binding energy.

## Introduction

**Fig. 1:**
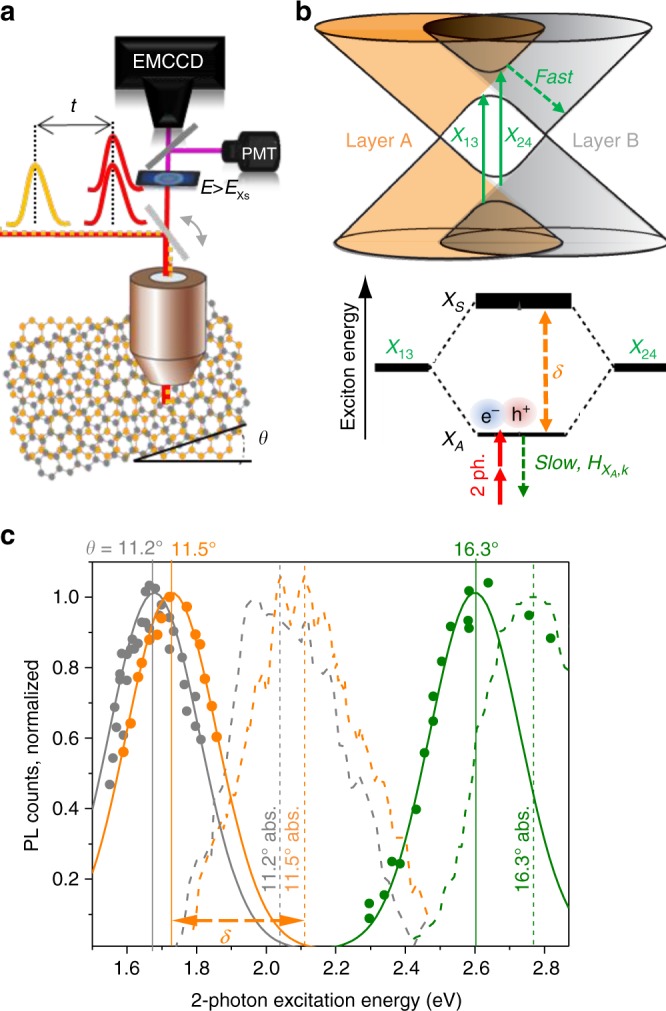
*θ*-tunable photoluminescence. **a** Setup for 2-photon PL microscopy on *t*BLG with filters passing *E* ≥ $E_{X_S}$. The delayed pulse, *t* is used only for transient absorption confirmation of the exciton binding energy. **b** Anticrossing band regions give two degenerate interlayer vHs transitions, *X*_13_ and *X*_24_. In an exciton model (lower panel): rehybridized transitions give a symmetric, bright (*X*_*S*_) and a 2-photon accessible antisymmetric (*X*_*A*_) states. **c** PLE peaks (circles) tune with *θ* = 11.2°, 11.5°, and 16.3°, and suggests 2-photon states, *X*_*A*_ lying *δ*-below dashed linear absorption peaks, *X*_*S*_

The interlayer orbital overlap in twisted bilayer graphene (*t*BLG) produce sharp optical absorption resonances that tune with the layer-stacking angle, *θ*^1–5^. Currently, there are two prevailing models explaining resonant light absorption in *t*BLG: the free-electron van Hove singularity (vHs) model, and the strongly bound-interlayer exciton model^1,6^. Figure 1b sketches the vHs model as two optically-allowed, degenerate transitions *X*_13_, and *X*_24_ between anticrossing regions of band-structure. This band-flattened region of *t*BLG has recently been associated with many surprising many-body electronic effects, such as exciton effects, *θ*-tunable superconductivity, and metal-insulator phase transitions^6–8^. In this work, we use 2-photon resonant excitation to find resonant PL emission from similar flattened-band interlayer states in *t*BLG.

Near *t*BLG band-structure anticrossing regions, theory predicts strongly enhanced electron-hole interactions from interlayer orbital mixing^6^. The lower panel of Fig. 1b outlines this alternative bound-exciton model with the renormalized vHs labelled as unbound, degenerate exciton transitions, *X*_13_ and *X*_24_. Symmetric and antisymmetric mixing of these two transitions is predicted to yield hybridized interlayer exciton states; specifically, an optically bright state, *X*_*S*_ and a strongly bound state, labeled *X*_*A*_. Using Bethe–Salpeter equation (BSE) simulations, Liang et al. predicted that the lower-lying exciton state, *X*_*A*_ is strongly bound, optically dark with vanishing exciton to graphene continuum coupling strength, $H_{X_A,k}$ (see Fig. 1b)^6^. To search for such optically dark interlayer exciton states in *t*BLG, we loosely assume parity-based optical selection rules from the hydrogenic exciton model and detect photoluminescence (PL) and excited-state absorption (ESA) signals generated via resonant 2-photon excitation^9–11^.

Using 2-photon PL, we can differentiate between the prevailing vHs model and the bound-interlayer exciton model in Fig. 1b. The vHs model for *t*BLG predicts only non-resonant hot-electron and blackbody PL emission because interlayer optical excitations thermalize rapidly (~10–20 fs) by the efficient electron-electron scattering in graphene^12^. We further estimate exciton binding energy, *E*_*b*_ using both 2-photon PL excitation (PLE) spectra and excited-state absorption (ESA) transient spectra methods. These figures of merit directly impact recent optoelectronic applications of *t*BLG such as chiral-light sensitive photosensors and light-induced metal-insulator phase transitions^13,14^.

## Results

### Weak *θ*-tunable interlayer PL emission

Non-resonant hot-electron and blackbody PL from both graphene and *t*BLG have been recently reported^15,16^. Unexpectedly, we observe PL emitted from *t*BLG domains after resonant 2-photon excitation. This weak emission is *θ*-tunable and independent of substrates and preparation method. To clearly observe resonant PL emission, we employ 2-photon excitation to remove the non-resonant scattering background with shortpass or bandpass optical filter stacks. The basic experimental setup showing a modified scanning confocal microscope is outlined in Fig. 1a. PL spatial maps and transient dynamics of single-domain *t*BLG were collected over our 0.3–1.8 eV laser tuning range. Both PL maps were acquired by raster scanning a ~1 μm wavelength-tunable laser spot across *t*BLG supported on silicon nitride or fused silica. The filtered 2-photon PL is collected with a back-illuminated electron-multiplying CCD camera or a photomultiplier tube (PMT) and lock-in amplifier.

Figure 1c (circles) plots PLE spectra collected after 2-photon excitation and the associated *θ*-tunable linear absorption spectra (dashed lines). The PLE spectrum is plotted after subtracting the non-resonant background signals such as blackbody emission^15,16^. Gaussian fits of the 2-photon PLE spectrum (solid lines) are peaked at *X*_*A*_ ≅ 2.61, 1.73, and 1.67 eV for the 16.3°, 11.5°, and 11.2° domains, respectively. Selective 10 nm wide bandpass filters were applied to confirm that the resonant PL emission was centered near ~2.7, 2.1, and 2.0 eV respectively. The domain angles were assigned by 1-photon linear absorption spectra which spectrally overlap with the corresponding 2-photon PLE peaks in Fig. 1c. Like PLE, the linear absorption spectra tune with stacking angle, *θ* but are blue shifted by an energy *δ*. In Fig. 1c, the 2-photon PLE and 1-photon absorption peak energies have a splitting, *δ* that is *θ*-tunable, ranging from *δ* = 160 to 380 meV (see Table 1). The absorption spectra after background-subtraction (*σ*_*tBLG*_ − 2*σ*_*G*_) are also used to assign the *θ*-orientation of the *t*BLG domains with about ±0.2° precision (see Supplementary Figs. 1–3)^4^.

**Table 1:** Exciton fine-structure resonances

*θ*	*X*_*S*_(eV)	*δ* (eV)	Δ(eV)	*E*_*b*_(*eV*)	Fig. ref. (method)
16.3^0^	2.75	0.16	0.34	0.51	3b (PL)
11.5^0^	2.10	0.37	—	>0.5	1c (PL)
11.2^0^	2.05	0.38	—	>0.5	1c (PL)
7.9^0^	1.55	0.34	0.33	0.69	4b (TA, blue)
7.9^0^	1.55	—	—	0.70	4b (TA, gray)

Three independent methods estimate the exciton binding energy *E*_*b*_ ≅ *δ* + Δ; the the peak splitting in 2-photon PLE, TA and intraband ESA spectra

### 2-photon PL spatial maps of *t*BLG domains

**Fig. 2:**
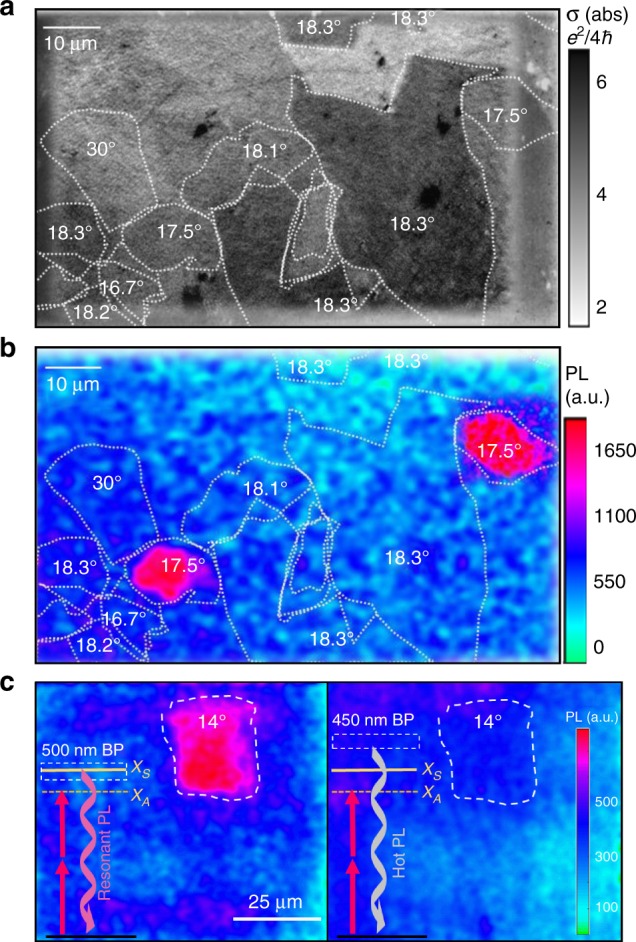
Maps of tBLG absorption and PL. **a** Linear absorption map taken at 2.9 eV excitation shows 20% resonant absorption enhancement of the 18.3° domains. **b** Scanning PL map of *t*BLG show resonant PL emission enhancement after 2-photon excitation (2 × 1.26 eV) of the 17.5° domains. The PL detection window is *E* > 2.65 eV. **c** PL emission wavelength is verified using bandpass filters to compare on-resonance PL (left, PL from *X*_*S*_) to off-resonance hot-PL (right) under identical excitation conditions

**Fig. 3:**
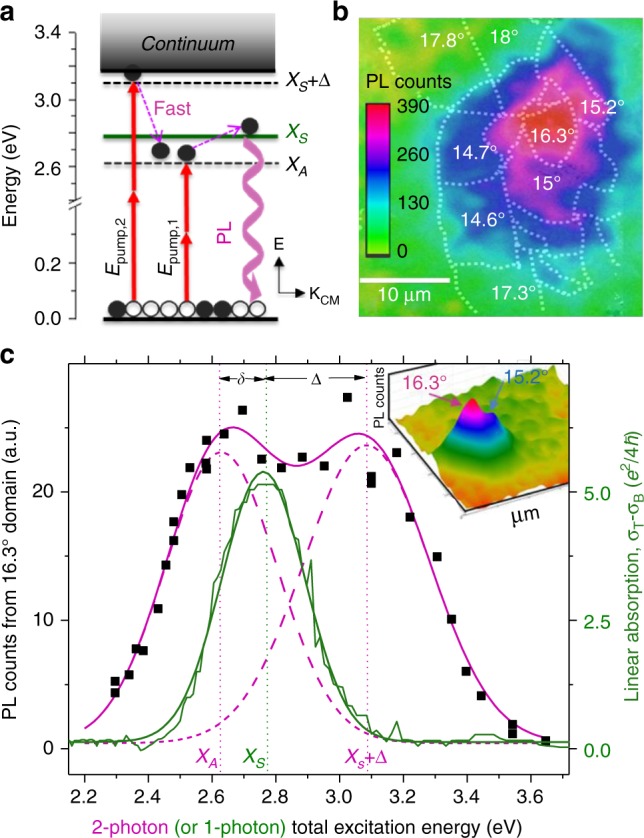
Interlayer exciton binding energy and PLE spectra. **a** Exciton model diagram showing emission of the *X*_*S*_ state after 2-photon excitation of *X*_*A*_ or continuum edge state, *X*_*S*_ + Δ. **b** Spatial PL map of CVD *t*BLG shows enhanced emission from the 16.3° domain and adjacent spectrally overlapping 15.2° domains after 2-photon excitation at 2 × 1.3 eV. **c** Extended range 2-photon PLE spectrum (black squares) fits to bimodal Gaussian peaks straddling the 1-photon linear absorption spectrum (green line). The total peak splitting *δ* + Δ, suggests *E*_*b*_ ≅ 0.5 eV. (Inset) 3D PL map showing ~5× PL resonant enhancement of the *θ* = 16.3° domain

The PL and absorption spectra plotted in Fig. 1c were compiled from full-frame spatial maps collected over spectrally tuned 1- and 2-photon excitation energies. For example, Fig. 2a shows a linear absorption contrast map with the 18.3° domains resonantly excited^4^. Figure 2b shows the corresponding *t*BLG spatial PL map collected at 1.26 eV excitation to search for allowed 2-photons transitions. Strikingly, two 17.5° domains show a ~5× enhancement of PL relative to the background hot-electron PL from the neighboring *t*BLG domains in Fig. 2b. Contributions below 2.65 eV were removed with an OD >6 filter stack. Selective bandpass optical filtering confirmed that emission energy matches the 1-photon absorption *X*_*S*_ resonance of the 17.5° domain at ~2.8 eV. This process showing resonant emission is detailed in Fig. 2c on 20 μm wide *θ* ~ 14° domain. This suggests that the resonant emission relies on electronic thermalization with the spectrally overlapping dark *X*_*A*_ state, as sketched in Fig. 3a. This bright–dark state splitting *δ*, was >150 meV, making *t*BLG emission inherently very weak.

### Verification of PL origin

To verify the signals in Figs. 1–3 are *θ*-tunable PL emitted from interlayer excitons, we must exclude any contributions or leaks from other resonantly-enhanced 2-photon scattering processes such as second harmonic generation (SHG) or 2-photon resonant Raman. 1-photon resonant Raman is well-documented in *t*BLG for *G*-band splitting with an energy of ~0.2 eV^4,17^. The unexpected Gaussian-like lineshapes of these early Raman studies provided the early clues that excitons may be involved in *t*BLG photophysics^4,18,19^. For Raman, the *G*-band spectral Stokes and anti-Stokes energies need to be a *θ*-independent^3,20,21^. However, our peak splitting energies vary strongly with *θ* from *δ* = 0.16–0.38 eV, ruling out a 2-photon resonant Raman signal origin.

Previously observed hot-electron PL was also excluded as the signal origin^15^. Such thermally drivenc emission produces a broadband blackbody emission spectrum, whereas we show in Figs. 2b and 3b that there is a ~5× PL enhancement over the non-resonant background signal collected from bilayer graphene. In Fig. 2c, we show that the PL emission collected with resonant vs. non-resonant bandpass (BP) filters give starkly contrasting emission instensity for large *θ* = 14° domain after resonant excitation. We conclude the majority of the photons are emitted resonantly rather than hot-electron or blackbody emission. Further examples of PL maps are in the Supplementary Figs. 3–5.

Both resonant 2-photon PL and transient absorption (TA) signals show a counter-intuitive linear trend with laser fluence. This trend is expected based on our concurrently measured 1-photon spectra showing clear square root ($\sqrt n$) pump-fluence dependence on carrier density, *n* (see Supplementary Fig. 7 for detailed fluence-dependent measurements). This square root is functionally distinct from graphene’s sub-linear Fermi-filling dependence^22^. Instead, $\sqrt n$ results from Auger or exciton–exciton annihilation. The net result requires a linear 2-photon PL flux-dependence, $I(n_{2ph}) \propto \sqrt {n_{2ph}^2}$ where *n*_2*ph*_ is proportional to the incident laser flux, which is typically 2 × 10^14^ photons/cm^2^ during 2-photon PL collection. As a control, we show in the supplement that our setup in Fig. 1a gives the typical 2-photon PL quadratic (*I* ∝ $n_{2ph}^2$) pump-power dependence when CdSe quantum dots were measured under identical experimental conditions. We further confirmed that the 1-photon TA signal in *t*BLG has a clear square root dependence, $I(n_{1ph}) \propto \sqrt {n_{1ph}}$ (see Supplementary Fig. 8)^23,24^. Very similar trends are reported for closely analogous materials such as carbon nanotubes (CNTs)^24–29^. This fluence dependence shows evidence that resonantly excited interlayer carriers undergo exciton–exciton annihilation.

### Estimates of interlayer exciton binding energy, *E*_*b*_

Using established 2-photon PL and intraband TA methods, we estimate *E*_*b*_ in resonantly excited *t*BLG^10,11^. Figure 3a shows how we measured *E*_*b*_ by expanding our energy range to include higher-lying 2-photon states predicted to cluster near the continuum edge for a hydrogenic exciton model. Upon 2-photon excitation at energy, Δ above the *X*_*S*_ absorption resonance with *E*_pump2_, the carriers will relax to the lower-lying dark state *X*_*A*_. 2-photon excitation at either *E*_pump1_ or *E*_pump2_ results in PL emission from the bright *X*_*S*_ state as the dark *X*_*A*_ and *X*_*S*_ state in Fig. 3c overlap spectrally. This bright–dark state splitting, *δ* quenches PL emission. A similar low-lying dark state is also thought to quench PL in CNTs^30^.

Figure 3b plots the 2-photon PL microscopy map of *t*BLG domains grown by chemical vapor deposition (CVD) and subsequently transferred to silicon nitride. Similar to the single-layer dry transfer *t*BLG in Fig. 2, we also show resonantly-enhanced PL (localized to the 16.3° domain). A partially overlapping absorption resonance results in the similarly stacked 15.2° domain emitting slightly-weaker PL. In Fig. 3c, the PLE spectral fits (magenta lines) for the 16.3° domain shows a bimodal distribution with two peaks spanning the 2.76 eV (green) absorption resonance at 2.61 and 3.10 eV. This spectral splitting energy is Δ ~ 0.34 eV, and this 2-photon PLE peak has almost twice the spectral width compared to the X_*S*_ or *X*_*A*_ peaks. This peak broadening may imply continuum edge mixing, suggesting we can estimate a lower bound for the exciton binding energy as *E*_*b*_ ≅ *δ* + Δ = 0.51 eV for the 16.3° domain shown. We summarize the *θ*–dependent peak splitting and *E*_*b*_ estimates in Table 1 below:

### Verifying *E*_*b*_ using ultrafast intraband ESA

The hot-electron vHs model in Fig. 1b has sufficiently predicted scattering resonances in Raman and circular dichroism^2,19,31,32^. For example,scattering in *t*BLG Raman and ARPES experiments do not optically populate interlayer excitons. Resonant PL emission is not conventionally observed for metallic materials because of strong screening of electron-hole pair interactions, and fast thermalization with continuum states. For resonant optical absorption, strong electron-hole interactions are already well-accepted in graphene-based systems. For example in single-layer graphene, there is ~0.4 eV red-shift in the absorption resonance about the *M* saddle-point owning to band renormalization from strong exciton effects^33,34^. Likewise, Ju et al. recently showed that 0° Bernal stacked bilayer graphene enables a bandgap of strongly bound interlayer excitons under high-fields and low-temperatures^8^. 1D metallic CNTs also have bound excitons with *E*_*b*_ ≥ 50 meV^35,36^.

Pump-probe kinetic studies and lineshape analysis of absorption and Raman spectra offered the early experimental motivation to search for interlayer exciton states in *t*BLG^6,17,23^. BSE simulations by Liang et al. support a strongly bound-exciton model for *t*BLG^6^. Theory simulates that the bound-exciton state, *X*_*A*_ has effectively zero electronic coupling with the lower metallic graphene continuum states^6^. Historically, such dark exciton states with $H_{X_A,k}$ = 0 (see Fig. 1b), have been termed ghost Fano resonances and observed in selected systems including some quantum dots^6,23,37^.

**Fig. 4:**
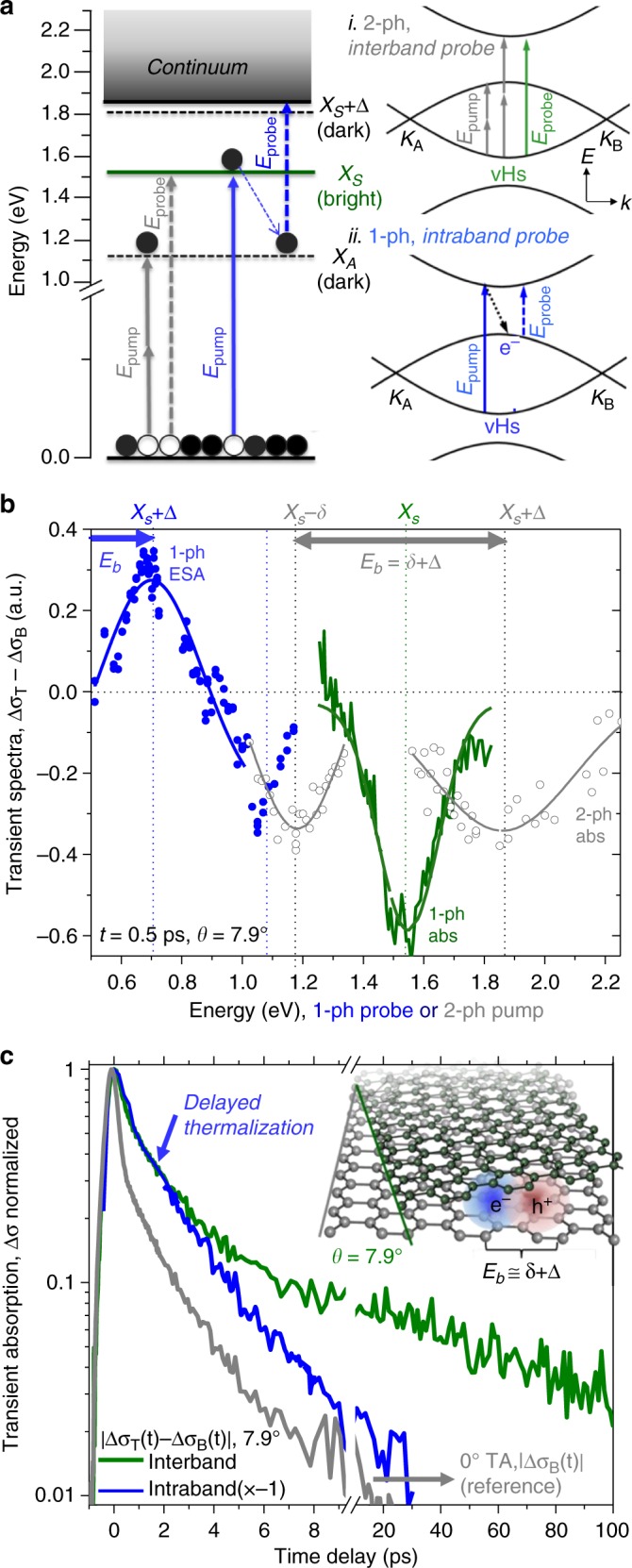
TA spectrum estimates of exciton binding energy. **a** Two measures methods of *E*_*b*_, blue arrows: pump *X*_*S*_, with probe ESA to intraband exciton band-edge states near the *e-h* continuum. gray arrows: 2-photon pump, probe at *X*_*S*_. (right inset) Equivalent experiments in a vHs model. **b** Blue and gray TA spectra independently estimate *E*_*b*_ at ~0.7 eV for *θ* = 7.9°. *t*BLG absorption spectra is in green. **c** Interlayer relaxation kintetics for both intraband and interband probe are delayed relative to the faster thermalization rate of 0° bilayer graphene shown in gray

In Fig. 4, we plot the transient spectra and exciton relaxation dynamics of a 7.9° *t*BLG domain by raster scanning two collinear ~160 fs pump and probe pulses. By measuring the differential TA of the probe pulse, we plot the full transient spectra at *t* = 0.5 ps in Fig. 4b. The corresponding relaxation kinetics are shown in Fig. 4c. To spectrally resolve the intraband exciton transition (dashed blue arrows in Fig. 4a), the pump beam was optically resonant at $E_{X_S}$ = 1.54 eV and the near-IR probe energy was scanned from 0.4–1.2 eV. The dynamics intrinsic to the interlayer states are isolated by subtracting the graphene 0° bilayer TA contribution (Δ*σ*_*B*_) from the *t*BLG TA response (Δ*σ*_*T*_) for both Fig. 4b, c^4^.

Transient spectra in Fig. 4b provide a secondary, independent estimate of *E*_*b*_. In analogous systems like CNTs, the exciton binding energy is estimated by an ESA spectrum of intraband exciton state transitions (see Fig. 4a, blue arrow)^38^. ESA transient absorption microscopy uses a NIR probe pulse to promote an exciton to the continuum band edge. The 2-photon selection rules are relaxed as this is an intraband transition within an exciton band of states^11^. In Fig. 4b, the TA spectrum (blue circles) is positively peaked at 0.7 eV, an ESA response. This suggests an intraband transition to the continuum edge-states illustrated in Fig. 4a, and that *E*_*b*_ ~ 0.7 eV for *θ* = 7.9° *t*BLG.

We verified the intraband ESA estimate of *E*_*b*_ by checking it with 2-photon TA microscopy (gray circles). Specifically, we scanned the 2-photon pump energy across the *X*_*A*_ and *X*_*S*_ + Δ state regions and probed the transient interband optical conductivity of the bright state *X*_*S*_. The resulting 2-photon absorption spectrum is shown in Fig. 4b (gray line). Two ground state bleach peaks are centered at 1.18 and 1.82 eV (gray line) and they straddle the 1-photon absorption peak at 1.54 eV (green line). Like the PLE in Fig. 3, the binding energy is estimated by the peak splitting, *δ* + Δ ≅ 0.69 meV. Both measurements methods shown in blue and gray in Fig. 4c gaive very similar estimates of *E*_*b*_. Our *θ*-tunable exciton binding energies are summarized in Table 1 and they are similar to the theoretically simulated binding energy value of 0.7 eV for a 21° *t*BLG domain^6^. Such large values from *E*_*b*_ are comparable to analogous semiconducting 1D and 2D excitonic materials^36,39–42^.

### Delayed thermalization of interlayer excitons

Lastly, we measure the interlayer carrier lifetimes in *t*BLG. In Fig. 4c, a least squares method is used to fit the decay of blue line to obtain an intraband lifetime of 1.05 ps that we associate with the exciton lifetime *X*_*A*_. The resonant interband (green line) *t*BLG carrier relaxation dynamics have been previously reported^23^. Comparing the two TA decay rates plotted in Fig. 4c (after subtraction of the much weaker, non-resonant 0° TA graphene response, Δ*σ*_*B*_), we note the absence of the fast initial electronic thermalization component typical for graphene (gray line). This suggests that interlayer *X*_*A*_ excitons may thermalize by exciton–exciton or exciton-phonon assisted processes, rather than by scattering from the electronic continuum^22^. Our observations of delayed thermalization of resonantly excited interlayer carriers for both the interband and the intraband kinetics supports the theoretical prediction that the bound-exciton has weak coupling to metallic continuum state ($H_{X_A,k} \cong 0$)^6^. Long lifetimes and predicted weak exciton-continuum coupling both suggests interlayer excitons may thermalize slower than they would in single-layer graphene.

## Discussion

In summary, we observe resonant, *θ*-tunable emission of PL upon direct 2-photon excitation of *t*BLG interlayer electronic states. Assuming hydrogenic exciton selection rules loosely apply, two-photon excitation was employed to optically excite dark exciton states^10^. Figures 2 and 3 show a ~5x resonant PL enhancement that we justify using a strongly bound-interlayer exciton model. This observation of resonant PL conflicts with the vHs model, as here electrons will thermalize rapidly to lower metallic continuum states by electron-electron scattering. Both CVD bilayer growths and dry-transferred stacked *t*BLG show similar PL emission in Figs. 2 and 3 for both the silicon nitride and fused silica substrates used. The PL is not surface quenched, suggesting it is emitted from protected interlayer electronic states.

As a 2D metal, *t*BLG interlayer excitons differ from excitons in similar materials like CNTs. For 2D metallic systems, unbound excitonic effects near the graphene *M*-point are well-known^31,32^. For *t*BLG, we suggest excitation of bound excitons are possible by mixing the two degenerate interlayer Fano resonances illustrated in Fig. 1b. Our estimates of the *t*BLG exciton binding energies are summarized in Table 1. We obtained *E*_*b*_, from the spectral peak splitting in both near-IR intraband TA and 2-photon PLE microscopy. The exciton binding energy scales with *θ* increasing from ~0.5 eV at 16.3° to 0.69 at 7.9°. This first measurement of *E*_*b*_ agrees with ~0.7 eV predictions from prior theory^6^. BSE theory predicts zero exciton-continuum coupling after the two interlayer degenerate transitions rehybridize. In accord, Fig. 4c (blue line) shows that interlayer excitons have much slower thermalization rate compared to 0° bilayer graphene (gray line). The ability to optically prepare interlayer excitons suggests *t*BLG is a 2D hybrid material with bound excitons coexisting alongside the metallic continuum states before decaying on a ~1.1 ps timescale. This unusual metal-exciton character of *t*BLG may enable stacking angle tunable materials applications from optically induced metal-insulator transitions to efficiency enhancements in graphene photosensing.

## Methods

### *t*BLG sample characterization

PL was observed from *t*BLG prepared both by CVD and the manual layer stacking by the dry transfer method^43,44^. The substrates were silicon nitride and fused silica and measurements were generally performed in ambient under a continuous nitrogen purge. 2-photon PL was observed for both the stacking methods and substrates used. The stacking angles of individual *t*BLG domains were assigned primarily with hyperspectral linear absorption microscopy after subtraction of the non-resonance graphene background (*σ*_*G*_)^4^. *θ*–stacking assignments were further verified by transient absorption (TA) and dark-field TEM^5,17,23^.

### 2-photon PL measurements

For the 2-photon PL and interband ESA measurements, we use a Coherent Chameleon (80 MHz, 140 fs) pumping an APE Compact optical parametric oscillator (OPO) with wavelength range 680–4000 nm. For the 2-photon PL microscopy measurements, the pump beam was generated by the OPO. The beam is then raster scanned by the piezo-scanning mirror and the 1-photon back reflection off the sample on an InGaAs detector provided a way to map the 2-photon and 1-photon responses concurrently. Total beam flux was 2 to 4 × 10^14^ photons/cm^2^. The emitted photoluminescence was measured with a TE cooled, back-illuminated EMCCD camera (ProEm HS, Princeton Instruments, 95% QE) and a Hamamatsu Si PMT as a secondary detection confirmation (with lock-in amplifier). To exclude scattered laser light from Rayleigh, Raman and residual second harmonic generation (SHG), collection of photons with energy less than twice the 2-photon excitation energy were excluded, calibrated Chroma or Thorlabs shortpass and bandpass optical filters stacks were used in front of the camera for emission detection to maintain an OD > 6 blockage throughout. All the measurements were performed at 295 K unless specified, in a nitrogen-purged box to minimize the chance of sample damage by oxidation.

### ESA and 2-photon transient absorption spectra

ESA TA experiments were used to probe intraband exciton transitions (Fig. 4, blue lines) using confocal transient absorption microscopy^23^. The pump beam was modulated at 1 MHz with an AO-modulator. Pump fluences were on average at ~10^14^ photons/cm^2^ with fluence dependence showing a square root exciton–exciton annihilation trend (see Supplementary Figs. 7 and 8).

Rigorous power normalization curves were taken in-situ for all spectrally resolved measurements. This includes the microscope objective transmission corrections, the spectral response of the detection system, and spectral characteristics of the optical filters were taken into account for each wavelength.

## Supplementary information

Supplementary Information

## Data Availability

The data that support the findings of this study are available from the corresponding author upon reasonable request.
