# Supplementary material for: Stacking angle-tunable photoluminescence from interlayer exciton states in twisted bilayer graphene
Source: Nat Commun. 2019 Mar 29;10:1445. doi: 10.1038/s41467-019-09097-x (PMC6441037; doi:10.1038/s41467-019-09097-x)
Supplement: Supplementary file 1 — Supplementary Information [file 41467_2019_9097_MOESM1_ESM.pdf]

Angle-tunable photoluminescence from interlayer exciton states in twisted bilayer graphene

Hiral Patel et al.

Supplementary Information:

Supplementary Note 1. Tight binding approximation: electronic band structure of *t*BLG: In *t*BLG, two graphene layers are rotated by an arbitrary angle with respect to each other. The rotation angle can be determined using dark-field transmission electron microscopy[1-6]. Graphene is known to have fast carrier relaxation down to the Fermi level. However, it was recently discovered that slower relaxation rate may be possible in metallic *t*BLG due to its unique band symmetry[1,26]. This has been shown theoretical modeling of the *e*-*h* interactions, and the simulations predict the existence of strongly-bound excitons in an otherwise metallic material *t*BLG[1].

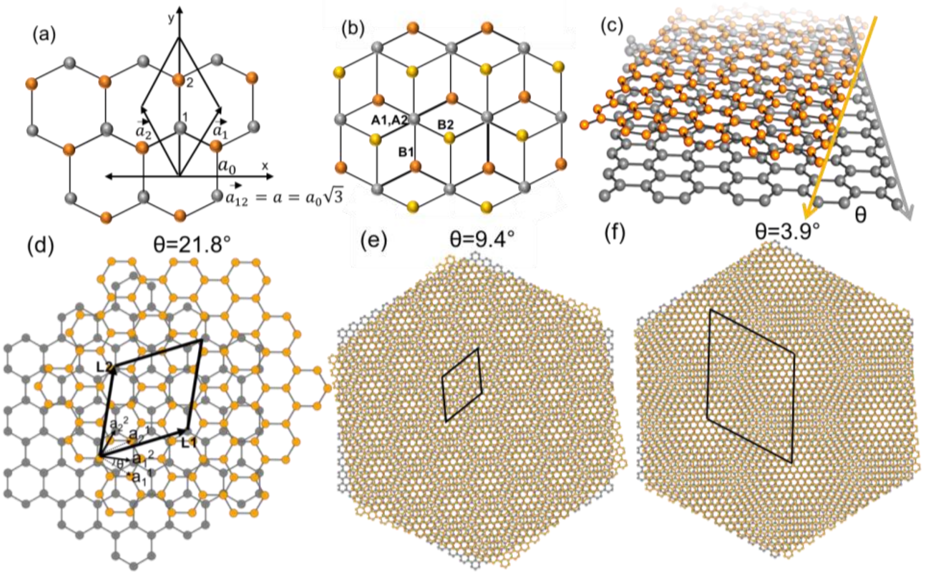

Supplementary Figure 1. (a) The lattice structure of single-layer graphene; Two carbon atoms per unit cell are labeled as 1, and 2 (grey, and orange). Primitive vectors are $\vec{a}_1$, and $\vec{a}_2$, (b) Lattice structure of bilayer graphene. Sub lattices for the lower layer are A1, and B1, and those for the top layer are A2, and B2, with relative positions of atoms projected onto the x-y plane (c) Lattice structure of twisted bilayer graphene for rotation θ, (d) *t*BLG θ=21.8°, (e) θ=9.4°, (f) θ=3.9°

The free electron graphene model considers the structure of twisted bilayer graphene defined by two layers of stacked bilayer graphene rotated around a common site by an arbitrary angle θ**.** The two lattice vectors for stacked bilayer graphene with $\theta = 0°$ are just $\mathbf{a}_1 = \mathrm{a}_0\ (1{,}0)$ and $\mathbf{a}_2 = \mathrm{a}_0 \left(\frac{1}{2}, \frac{\sqrt{3}}{2}\right)$. For non-zero angles the lattice vectors are more complicated because they must span the new superlattice, and are given stacking angle indexed vectors; $\mathbf{a}_1^1 = \mathbf{R}\left(-\frac{\theta}{2}\right)$, and $\mathbf{a}_1^2 = \mathbf{R}\left(\frac{\theta}{2}\right)$**.**[17-20] Rotation angle $\theta = 0°$ produces AA stacked bilayer graphene and $\theta = 60°$ corresponds to AB bilayer graphene stacking.[17]

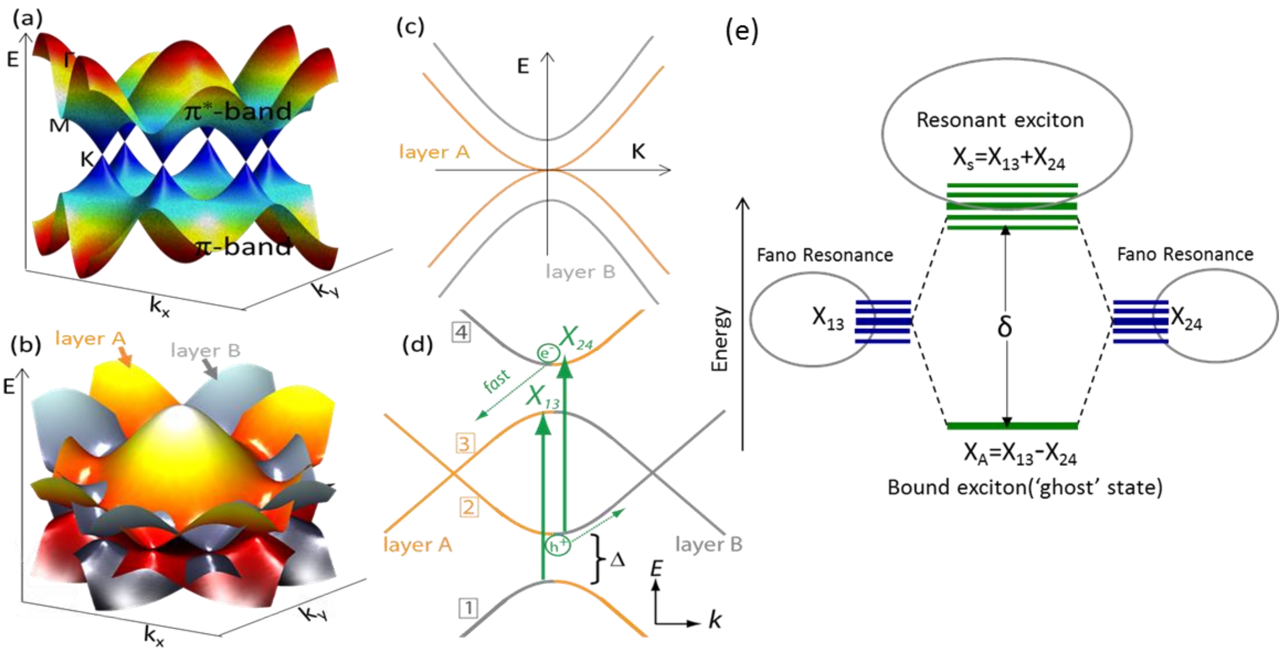

Supplementary Figure 2. Tight-Binding Model band picture: (a) Representation of the band structure for single layer graphene. (b) *t*BLG free electron band-structure, (c) the band dispersion near the K-point in bilayer graphene, (d) cross-sectional view of *t*BLG shows vHs transitions X_{13}, X_{24} between avoided crossing regions. (e) **Interlayer exciton picture**: Upon resonant optical excitation, degenerate Fano resonance states X_{13} and X_{24} rehybridize producing a higher energy resonant exciton state X_s and lower energy bound exciton state X_A.

Rotation angle θ and $-\theta$ produce a similar band structure. *t*BLG does not have a periodic structure in general because of mismatch between the uniformity of layers with respect to each other. The structure is periodic only for some special angles with a well-defined unit cell. A periodic lattice structure of *t*BLG for rotation angles $\theta = 21.8°$, and $\theta = 9.4°$, $\theta = 3.9°$ is shown in Figure Supplementary Figure 1d, e and f. When the rotation angle is small, the interference between two lattice vectors in two layers gives rise to the pattern called the 'Moiré pattern'[18-20]. The unit cell is relatively big for small rotation angles as seen in Supplementary Figure 1e and f.

Supplementary Note 2. Review of bound exciton model for *t*BLG:

Upon photoexcitation of *t*BLG, exciton effects may manifest themselves owing to Coulombic interactions of e-h pairs. To theoretically model the many-body excitonic effects, it is necessary to solve the BSE as shown by Rohlfing et al.[5]: $(E_{ck} - E_{vk})A^S_{vck} + \sum_{v'c'k'}\langle vck|K^{eh}|v'c'k'\rangle A^S_{v'c'k'} = \Omega^S A^S_{vck}$, where, A^S_{vck} is the exciton wave function in k-space, $\Omega^S$is the exciton eigenenergy, $K^{eh}$is the e-h interaction kernel, and $|vk\rangle, |ck\rangle$ are the hole and electron states, respectively. In order to predict possible bound exciton states, it is important to know the *e-h* attractive energy E^S_a, which roughly corresponds to the binding energy, and recently the first-principle BSE simulations in Liang et al.[14] reported that $E^S_a = 0.5\ eV$ for 21.8° *t*BLG.

Specifically, Liang et al. solved for $E^S_a = \langle S|K^{eh}|S\rangle = \sum_{vck}(E_{ck} - E_{vk})|A^S_{vck}||A^S_{vck}| - \Omega^S$, which is the net exciton kinetic energy plus the weighted single-particle band energy difference between electrons and holes[1,10,14]. To simplify the direct analysis of the first principle BSE simulation, low-energy effective model is often further assumed, giving a simpler perturbed Hamiltonian that is analogous to Equation (1), specfically[10];

$$H(k) = \begin{pmatrix} H_0(k,0) & T^+ \\ T & H_0(k-\Delta K,\theta) \end{pmatrix}, \text{ with } H_0(k,0) = \hbar v_F \begin{pmatrix} 0 & e^{-i\theta}(k_x - ik_y) \\ e^{i\theta}(k_x + ik_y) & 0 \end{pmatrix}, \text{ where } T = \Delta\begin{pmatrix} 0 & 1 \\ 1 & 0 \end{pmatrix} \quad (1)$$

The average interlayer interaction between AB and BA stacking order is described by matrix T with interlayer coupling strength Δ. The low-energy effective model is best for small-twisting angles because the linear Dirac-Fermion dispersion

is preserved. Using the BSE and the low effective model, Liang et al.[1] found that upon resonant excitation of *t*BLG, you can access degenerate Fano resonant transitions X_{13}, X_{24} which then effectively rehybridize as depicted in Supplementary Figure 2c. The symmetric superposition of the two degenerate transitions gives rise to a higher energy bright state and anti-symmetric superposition give rise to the lower energy dark state ('ghost' state, see Supplementary Figure 2e).[12] The constructive interference couples with the lower lying continuum which broadens the resulting resonant bright excitonic state. The antisymmetric superposition cancels the coupling between the lower lying continuum making it a localized bound excitonic state. The so-called ghost Fano resonance effect was found in quantum dot molecules.[12] This model is appropriate for small twist angles, however, the double resonance of transitions and the destructive interference plays a crucial role in the existence of strongly bound excitons.[1,14]

Supplementary Note 2.1. Idealized 'Textbook' Ghost Fano Resonance Effect: A Ghost Fano resonance effect has been predicted for resonant tBLG optical excitation by first-principle GW-BSE simulations.[1] Qualitatively, this effect can be understood through two-degenerate transitions (Fano resonance) causing destructive interference that leads the elimination of the exciton-continuum coupling term. There is an equivalent textbook explanation explaining the base concept of how degenerate overlap of excitonic transition can cause the exciton-continuum coupling term o vanish, i.e. $H_{12,k}=H_{Ex,k}=0$.[1] Specifically, if we write the total Hamiltonian of our system as $H_{tot} = H_{12} + H_k + H_{12,k}$ the bound-exciton basis Hamiltonian, H_{12} can be re-expressed by mixing the two degenerate transitions, X_{13} and X_{24} to give $H_{12} = \varepsilon_c (b_1^\dagger b_1 + b_2^\dagger b_2) + \delta (b_1^\dagger b_2 + b_2^\dagger b_1)$ in terms of raising and lower operators of the ground and excited exciton levels. The continuum of graphene states sums over all states, *k*, and can be expressed as $H_k = \sum_k \varepsilon_k (c_k^\dagger c_k)$. By analogy, we can further treat the coupling of the symmetric and antisymmetric exciton states to the continuum states as $H_{12,k} = \sum_{k,i=1,2} A_k (c_k^\dagger b_i + b_i^\dagger c_k)$.

A standard textbook question is then to ask how the coupling of the continuum states couple to the exciton states, i.e. what is $H_{12,k}\left|X_A\right\rangle$ where $\left|X_A\right\rangle = \frac{1}{\sqrt{2}}\left(\left|X_{13}\right\rangle - \left|X_{24}\right\rangle\right)$ is the anti-symmetrized exciton state with energy (in the uncoupled exciton basis) calculated by $H_{12}\left|X_A\right\rangle = (\varepsilon_c - \Delta)\left|X_A\right\rangle$. Likewise, it is shown below that an anti-symmetrized mixing of degenerate states give $H_{12,k}\left|X_A\right\rangle = 0$, provided that the optically prepared state mix with degenerate exciton-like states (i.e. the Fano resonance X_{13} and X_{24}). The net result causes the energy perturbation from the exciton-continuum coupling to vanish, as shown below,

$$
\begin{aligned}
H_{12,k}\left|X_A\right\rangle &= \frac{1}{\sqrt{2}}\left(\sum_k A_k (c_k^\dagger b_1 + b_1^\dagger c_k)\left|X_{13}\right\rangle - \sum_k A_k (c_k^\dagger b_2 + b_2^\dagger c_k)\left|X_{24}\right\rangle\right) \\
&= \frac{1}{\sqrt{2}}\left(\sum_k A_k \left|k\right\rangle - \sum_k A_k \left|k\right\rangle\right) \\
&= 0
\end{aligned}
$$

The above calculation is highly idealized. Liang et al. shows this basic premise of zero-coupling to the continuum also applies to the extended unit cell of twisted bilayer graphene by using full GW-BSE.[1]

SUPPLEMENTARY METHODS

i. Samples & Stacking Angle Determination: *t*BLG was obtained by both 'as grown' CVD method (sample shown in Fig. 3) and by artificially transferring single layer grown graphene layers (sample shown in Fig. 2ac). For artificially transferred *t*BLG, graphene was grown with an aligned CVD growth method on a copper substrate**.** The copper foil with graphene is then cut into two pieces and is then transferred in a *t*BLG configuration to a SiN or fused silica substrate with the artificially transferred technique. This technique can result in large domain *t*BLG material. The samples were characterized using the combination of hyperspectral absorption, transient absorption, and dark field TEM to locate twisted domains.[8] The final angle assignment of the domains was done using well-established methods looking at linear absorption peak energies. The *t*BLG samples were prepared with *as grown CVD* or artificially stacked method and they are both environmentally p-doped as determined by point Raman spectral shifts shown in Supplementary Figure 3c. The G peak location suggests a doping level on the order of 10^{12} to 10^{13} cm^{-2}. The sample to sample doping variation can be

on ~10^{12} level due to doping caused by the transfer process. This paper further shows that small changes in the doping (CVD vs. dry transfer stacked), and dielectric environment (e.g. three different substrates silicon nitride, fused quartz and silicon) all show similar PL and/or delayed thermalization kinetics (see main text).

Two-photon PL and TA microscopy on *t*BLG requires (i) identification of large-area *t*BLG regions (ii) -beam diffraction limited resolution TA. Identification begins using diffraction-limited scanning confocal TA microscopy. The correspondence between absorption resonance and the twist angle has been well previously established.[2,3] Once all optical measurements were complete, darkfield transmission electron microscopy (TEM) was used to determine precise angle assignments. After the regions with anomalous electronic relaxation dynamics are identified, the precise absorption resonances were later measured using hyperspectral absorption imaging. By collecting the full-frame absorption movies vs. wavelength, specific absorption spectra can be acquired by integrating over a defined region and plotting as a function of absorbing wavelength. Similar results were obtained for *t*BLG on silicon, silicon nitride, and fused silica substrates used for PL detection.

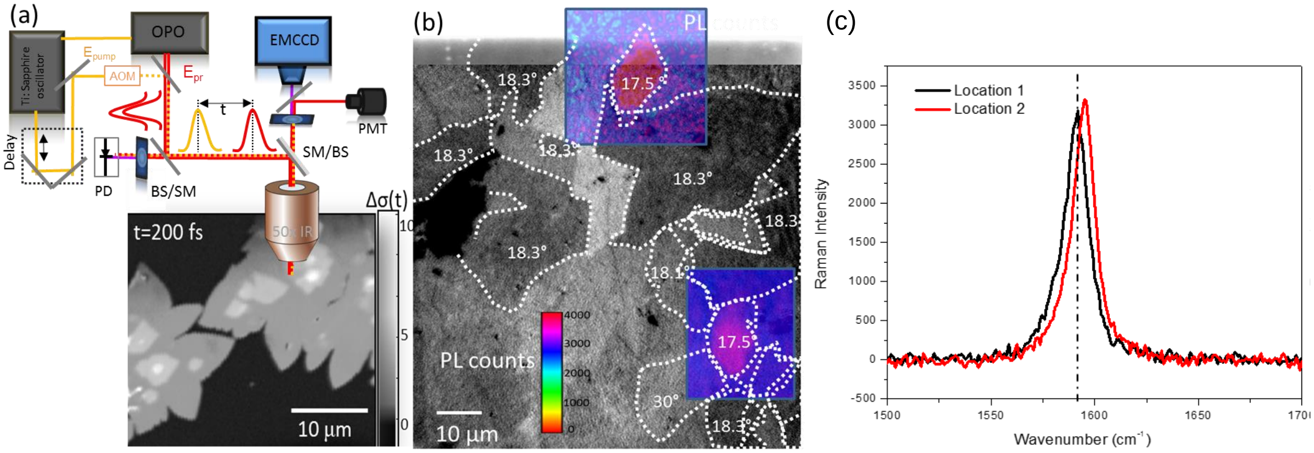

Supplementary Figure 3. **(a)** Optical setup for transient absorption 2-photon PL and TA microscopy. Sub-diffraction limited pump, and probe pulses are raster scanned, and the sample can be imaged against the laser noise floor by employing a lock-in amplifier detection synched to an acousto-optical (AO) modulator (1 MHz). **(b)** Example overlay of resonant 2-photon PL resonant at 17.5° on a linear absorption map resonant at 18.3°. **(c)** Shifts in G-band resonance of point Raman taken from graphene sample growth used in this paper. These shifts can be used to approximate p-doping concentration variation over the the samples measured in the main text.

ii. Excited state transient absorption of intraband exciton transitions (Fig 4 in manuscript): For the excited state absorption experiment and 2-photon photoluminescence measurements, synchronized Ti: Sapphire oscillator (Coherent Chameleon Ultra II) with wavelength range 680 - 1080 nm, 80 MHz repetition rate and optical parametric oscillator with wavelength range 1000 - 4000 nm was used. Excited state absorption (ESA) experiment was done with confocal transient absorption microscopy.[26] A collinear pump and a probe beam were obtained with the synchronized Ti: Sapphire oscillator with the OPO. The pump beam was modulated at 1 MHz with an AO-modulator (Gooch and Housego). The probe fluence power was ~5% of the pump power. For the pump power dependence measurements, the probe power was fixed at (~1 x10^{12} photons/cm^2). The beams were aligned to the mechanical delay stage, then raster scanned by a piezo-scanning mirror and then coupled to a confocal scanning microscope with a 50X-IR objective, NA = 0.65. 140 fs FWHM pulse duration was measured by the cross-correlation of the pump and probe under the objective. The spot size of the pump and the probe beams were measured to be ~1.5 µm using a gold pad features by fitting the reflection profile. A thermoelectrically cool (TE) cooled InGaAs detector connected to Zurich HF2LI lock-in amplifier was used to detect the transient absorption response. Appropriate optical filters in front of the detector were used to let only the probe beam through while blocking the pump beam.

iii. 2-Photon photoluminescence (PL) measurement details and supplemental data: For the 2-ph photoluminescence microscopy measurements, the pump beam was obtained either through oscillator or OPO. The beam is then raster scanned by the piezo-scanning mirror and then coupled to a microscope with an Olympus 50X-IR objective, NA = 0.65. The back reflection of the sample was obtained with an InGaAs photodetector. Long pass optical filters were used in the line before

the microscope to block possible 2ω light from the laser source. The emitted photoluminescence was measured with a thermoelectrically cooled, back-illuminated EMCCD camera (ProEm HS 1024x1024, Princeton Instruments) and a Hamamatsu Si PMT was also used a secondary detection confirmation. Short pass and bandpass optical filters were used in front of the camera for emission detection. All the measurements were performed at 295 K unless specified typically under dry nitrogen purge shown in Supplementary Figure 5a. Microscope objective transmission corrections, the spectral response of the detection system, and spectral characteristics of the optical filters were taken into account for each wavelength. Supplementary Figure 4 and 5 show resonant PL maps from various *t*BLG domains with different stacking angles. Supplementary Figure 5 examines wavelength dependence of the emission and show the PL-window response of a large artificially stacked CVD domain of θ~14°. Comparing Figures Supplementary Figure 5 d to f the 2-photon PL signal can only be detected when the exciton is resonantly excited and detected. This is consistent with the interlayer bound-exciton model, not hot PL.

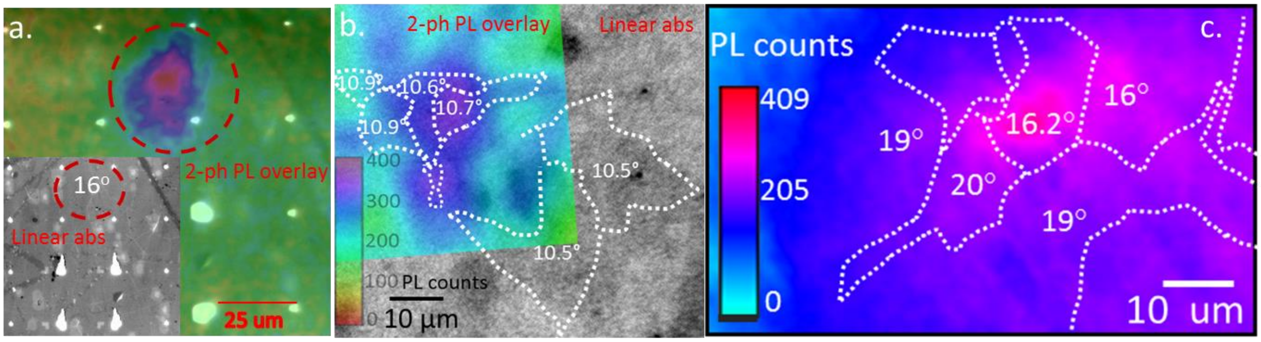

Supplementary Figure 4: Examples of resonant PL maps from other *t*BLG grains not shown in the main text. All show a 4x to 7x PL resonant enhancement over the graphene hot-electron background. **(a)** PL map (16° grain) overlaid with transparency on the linear absorption map (*inset*) at 2.3 eV. **(b)** Low angle PL map overlay showing PL at a 10.7° excitation resonance. **(c)** PL map overlay showing emission at 16.2° excitation resonance.

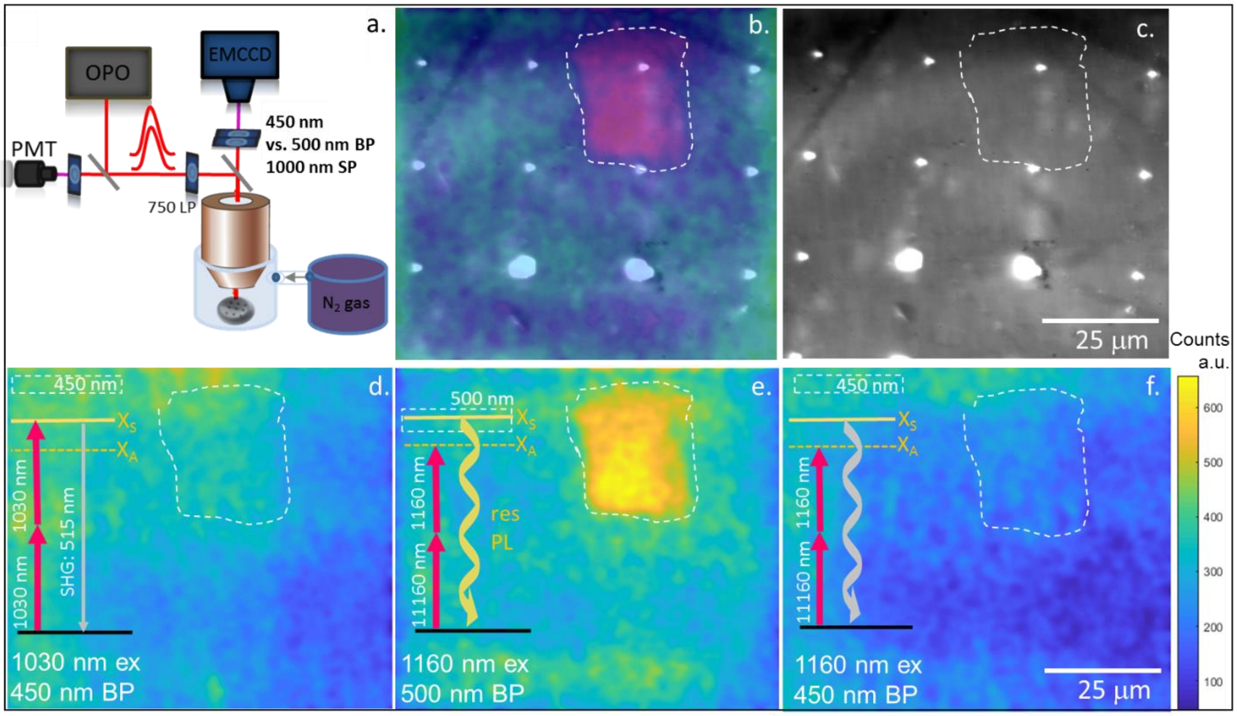

Supplementary Figure 5- Spectral emission map wavelength dependence. **(a)** Experimental setup to prove resonant emission involves changing bandpass filters regions. Weak 2-photon resonant emission maps are collected on an EMCCD,

after 1-photon, and 2-photon SHG scattering is filtered out **(b)** Overlay of the PL emission map of a ~14° *t*BLG domain and camera image of the fused silica substrate with alignment markers. **(c)** Corresponding substrate optical camera image with an outline of the 14° domain with optical resonance of about 505 nm. **(d)** 2-photon exciton of bright state does not enhance hot-PL, consistent 1-photon selection rules, no hot PL is observed. **(e-f)** In particular, we compared the difference between a 500 nm (10 nm width) bandpass filter centered on the bright X_S absorption resonance and a 450 nm filter (10 nm width) lying above. Comparing e and f, we can discern strong resonant PL emission. This suggests the signal is resonantly emitted near its 505 nm absorption resonance and cannot be hot-PL emission.

Supplementary Note 3: Transient Optical Conductivity

Supplementary Note. 3.1: the intralayer transient optical conductivity

In transient absorption microscopy, we measure the change in the amplitude of the reflected probe beam at a probe energy E_o, in the absence (R_2) and presence (R_1) of a pump beam. Under optical excitation conditions, transient reflectivity is related to the optical conductivity, σ_i by[4,7]:

$$\frac{\Delta R(t, E_o)}{R} = \frac{R_2 - R_1}{R_1} = \frac{4}{n_s^2 - 1}\frac{4\pi}{c}\sum_i \mathrm{Re}\left[\sigma_i(E_o, T_e(t)) - \sigma_i(E_o, T_l)\right]$$

Both interband and intraband processes contribute to the total optical conductivity,

$$\sigma_{TOT} = \sigma_{inter} + \sigma_{intra}$$

When the Fermi populations are evaluated (to first order) it has been shown that[3,7]:

$$\frac{\Delta R(t)_{inter}}{R} \propto \tanh\left(\frac{E_o \pm E_F}{2k_B T_e(t)}\right) - \tanh\left(\frac{E_o \pm E_F}{2k_B T_l}\right) \qquad (2)$$

$$\frac{\Delta R(t)_{intra}}{R} \propto \frac{\Gamma k_B T_e(t)}{(E_o \pm E_F)^2 + \Gamma^2} - \frac{\Gamma k_B T_l}{(E_o \pm E_F)^2 + \Gamma^2} \qquad (3)$$

The absolute sign of the intraband and interband transient signals are opposite, permitting experimental separation.[7] We attribute the bleach to the transient interband optical conductivity, and the excited state absorption (ESA) to intraband transitions. As observed in previous works (e.g. ref 7), we observed that intraband contribution is best determined by its absolute sign (under the specific sample and carrier densities used in our manuscript). For probe energies <1.3 eV, the relative intraband kinetic contribution is weak, and can be approximately ignored and we can approximately remove the single layer contribution for the TA kinetics by plotting $\Delta\sigma_{tBLG}(t)$-$\Delta\sigma_{bBLG}(t)$ as we do in Fig. 4c of the paper.

Supplementary Note 3.2: the interlayer transient optical conductivity; interband bleach vs. intraband ESA

Currently, there exists no closed-form expression for the transient optical conductivity near the *t*BLG optical absorption resonance. In the limit that the two layers of graphene are electronically decoupled, the transient optical conductivity, $\Delta\sigma(t)$ is obtained by evaluating for the Fermi-Dirac electronic population at the desired energy. Generally, the relative magnitude of a pump-probe or transient absorption (TA) response is given by:

$$\Delta\sigma(t, E_{pr}) \propto n(t)\left[\sigma_{ESA}(E_{pr}) - \sigma_{SE}(E_{pr}) - \sigma_o(E_{pr})\right] \qquad (4)$$

where $n(t)$ is the electronic carrier population occupying the probed state energy(E_{pr}). σ_{ESA}, σ_{SE} and σ_o are the absorption cross-sections of the excited state absorption (*ESA*), stimulated emission (*SE*) and the ground state spectral bleach at an incident probe energy, E_{pr}. The ESAs (e.g. intraband absorption in graphene) gives the opposite (negative) $\Delta\sigma(t)$ sign, while the "ground state bleach" response is positive, arising from interband Pauli blocking effects.

In the free carrier model, Pauli blocking refer to photoexcited holes or electrons lying at the same energy of the probe beam, leading to decreased absorption (positive signal by our convention). In the bound exciton model, Pauli blocking chiefly refers to decreased probe beam absorption form a depleted ground exciton band. Excitons tend inherently have very narrow electronic distribution in their center of mass momentum space, $K_{cm} = (k_e + k_h)/2$, enabling us to approximate treat the ground and excited states as a discrete energy broadened by static and dynamic environmental interactions. In such a bound-exciton model, the carrier density in the above equation for $\Delta\sigma(t)$ decays approximately exponentially in time, according to the rate equation[28]:

$$\frac{dn}{dt} = P\delta(t) - \frac{n}{\tau} \qquad (5)$$

where, P is the incident photon flux, $\delta(t)$ approximates our 140 fs excitation pulses, and τ is the exciton relaxation lifetime of the (X_{13}-X_{24}) state. If there are multiple states (e.g. X_{13}-X_{23} and X_{13}+X_{23}) or multiple relaxation pathways there will be multiple lifetimes τ_1 and τ_2 in the rate law.

In Supplementary Figure 6a, we show further transient absorption spectra to Supplementary Figure 4b,c. This plot shows raw unscaled TA spectral data. We believe negative signal in Supplementary Figure 6b is most likely an artifact common to TA spectra. The details of this phenomenon is beyond the scope of this article (see ref. 5 for example).[5] There remains some possibility that the unlabeled negative signal may also be ground state bleach recovery of the exciton state because its energy is very close to the dark sate exergy, X_s. in fig 4b (gray). Resolution of such details will be reseverved for future TA-focused work.

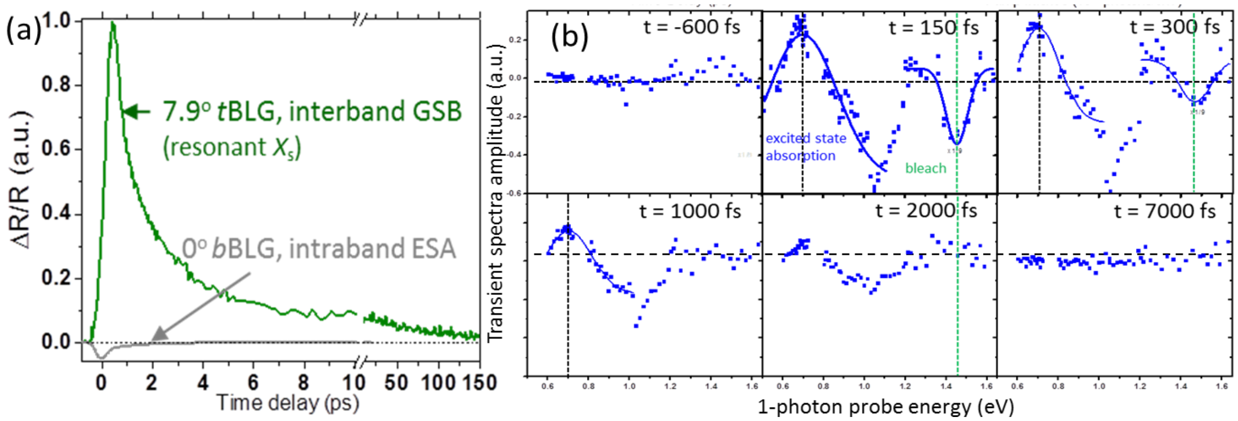

Supplementary Figure 6. (a) Normalized relaxation dynamics for 1-photon pump and probe (green) resonant with 7.9° *t*BLG showing an absolute signal scaling relative to a Bernal stack domain. **(b)** TA spectral evolution (corrected for the 0° graphene background contribution) for -0.6 to 7 ps delays, after a 1-photon pump near-resonant with a 7.9° domain region.

Supplementary Note 4: Multi-Photon Power Dependence & Nonlinear Kinetic Response

Observed non-linear relaxation kinetic model:

The 2-photon carrier generation term grows quadratically with laser fluence, I ($G \sim I^2$). This quadratic dependence is shown in Supplementary Figure 7 under identical conditions for a standard quantum dot fluorophore. However, in tBLG only one single domain is coherently excited creating multiple excitons on resonance. Consequently, the overlapping excitons annihilate (widely observed in semiconducting carbon nanotubes and 2D transition metal dichalcogenides).[27] This has previously demonstrated with pump-probe measurements.[27] Therefore, the kinetic rate for the excitonic population may be approximated as:

$$\frac{dn}{dt} = G\delta(t) - \gamma n^2 - kn \qquad (6)$$

where k is the sum of radiative and non-radiative linear rates, and G is the laser-induced carrier generation term. γ is the nonlinear annihilation term, chiefly interlayer exciton-exciton annihilation (an Auger process).

We have shown in Supplementary Figure 7-8 that multiple excitons are created on the same *t*BLG domain, and the γ-term dominates the fluence-dependent PL and TA measurements.[23] In the commonly applied quasi-static approximation one finds that, $n_o \propto \sqrt{G}$. This says the total number of carriers remaining to emit PL scale with square-root of the laser power for the 1-photon process (see green, pump-probe Supplementary Figure 7). Therefore, for a 2-photon process, the carriers are populated as $G \sim I^2$, and so the PL scaling must be then linear for a 2-photon process ($n_o \propto I \propto \sqrt{n_o^2}$).

Experimental pump-fluence dependence on PL: We did detailed power-dependence on all measurements. The predicted 1-ph PL response for *t*BLG is $n^{1/2}$ (as also seen in CNTs) and the observed 2-ph response PL is $(n^2)^{1/2}=n$. To check this was reasonable, we measured 2-photon CdSe quantum dot PL alongside to prove it had the expected n^2 dependence.

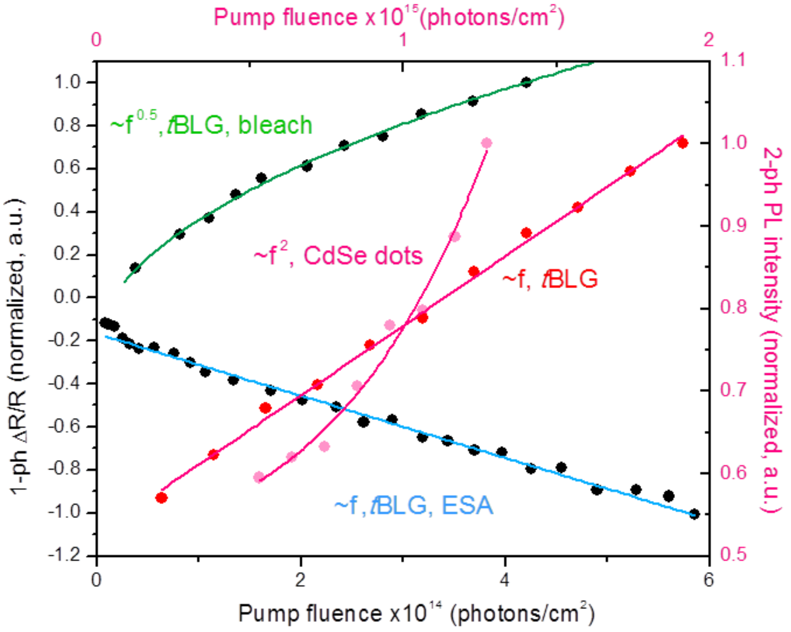

Supplementary Figure 7. Power dependence of 1-photon TA measurements (left axis) and 2-photon PL (right axis) taken *in situ*. Non-linear annihilation processes are evident in both the TA and PL data.

Experimental pump-fluence dependence on TA kinetics: Supplementary Figure 7 and 8 show that Auger annihilation contributes strongly to the TA power dependence of *t*BLG. Auger exciton annihilation effects must be including the electron dynamics of strongly-bound excitonic systems such as CNTs, with the following characteristic kinetics:

$$\frac{dn}{dt} = P\delta(t) - \frac{n}{\tau} - \frac{\gamma n^2}{2} \quad (7)$$

Here, P is the incident photon flux, δ(t) approximates our 140 fs excitation pulse, τ is the exciton relaxation lifetime of the (X_{13}-X_{23}) state, and γ the exciton annihilation rate constant. In the short-time limit, when exciton density is highest, the above equation may be approximated as, $n_o \sim (2P/\gamma)^{0.5}$. This suggests that if the *t*BLG TA signal comes for bound exciton states, it may scale with the square root of photon flux for a one-photon excitation, and linearly for two-photon excitation. Accordingly, for *t*BLG, we observe in Supplementary Figure 8 ii a characteristic square root amplitude dependence that is seen in analogous systems like the SWCNTs. Consistent with dominant exciton annihilation, we find the two-photon *t*BLG response grows only linearly with photon flux.

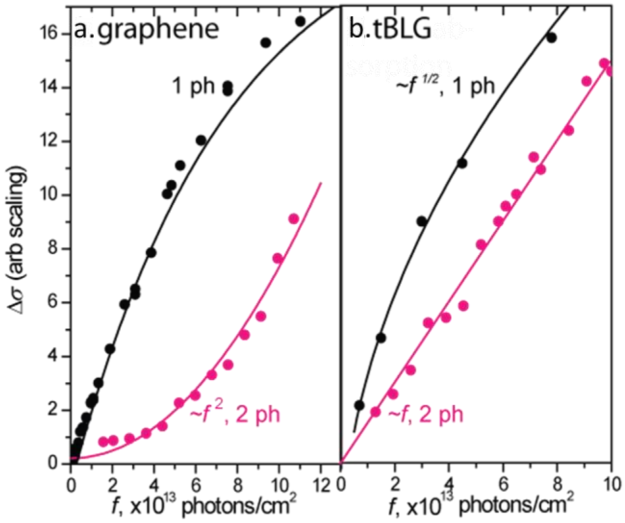

Supplementary Figure 8. Transient absorption signal vs. photon flux, *f*. **a.** Graphene, showing Fermi-Dirac and quadratic fits. **b.** *t*BLG TA, showing square root function and linear fits.[26]

As a control, we also show in Supplementary Figure 8a, the single-sheet graphene interband TA power dependence fit to graphene's characteristic Fermi-Dirac electronic filling function. Conversely, the two-photon TA fits to a quadratic function for delay times near *t*=0, as required. We conclude underlying square root behaviors observed suggest Auger annihilation processes in *t*BLG. This further supports a bound exciton model, and suggesting future parallels between *t*BLG and s-SWCNTs photophysics, motivating future investigations.

Supplementary References

[1] Y. Liang, R. Soklaski, S. Huang, M. W. Graham, R. Havener, J. Park, and L. Yang, Physical Review B, 2014, 90(11), 115418.

[2] R. W. Havener, C.-J. Kim, L. Brown, J. W. Kevek, J. D.Sleppy, P. L. McEuen, and J. Park, Nano Letters, 2013, 13(8), 3942.

[3] R. W. Havener, H. Zhuang, L. Brown, R. G. Hennig, and J. Park, Nano Letters, 12(6), 3162-3167, 2012

[4] M. Breusing, C. Ropers, and T. Elsaesser, Phys Rev Lett, 102(8), 2009

[5] M. Rohlfing and S. G. Louie, Physical Review B 62(8), p. 4927, 2000.

[6] M.W. Graham, S. Shi, D. Ralph, J. Park, P.L. McEuen, Nature Physics, 9, 103, 2013

[7] L. Malard. K.-F. Mak, A. Castro, N. Peres, and T.F. Heinz, New Journal of Physics, 15, 015009, 2013

[8] S. Winnerl ,M. Orlita, P. Plochocka, P. Kossacki, M. Potemski, T. Winzer, E. Malic, A. Knorr, M. Sprinkle, C. Berger, W. A. de Heer, H. Schneider, and M. Helm, Phys Rev Lett, 107, 237401, 2011.

[9] Y. Zhang, T.-T. Tang, C. Girit, Z. Hao, M. C. Martin, A. Zettl, M. F. Crommie, Y. R. Shen, and F. Wang, " Nature 459(7248), pp. 820–823, 2009.

[10] E. J. Mele, Physical Review B 84(23), p. 235439, 2011.

[11] E. V. Castro, K. Novoselov, S. Morozov, N. Peres, J. L. dos Santos, J. Nilsson, F. Guinea, A. Geim, and A. C. Neto, Journal of Physics: Condensed Matter 22(17), p. 175503, 2010.

[12] M. L. De Guevara, F. Claro, and P. A. Orellana, Physical Review B 67(19), p. 195335, 2003

[13] A. Kuzmenko, I. Crassee, D. Van Der Marel, P. Blake, and K. Novoselov,, Physical Review B 80(16), p. 165406, 2009.

[14] H. Min, B. Sahu, S. K. Banerjee, and A. MacDonald, Physical Review B 75(15), p. 155115, 2007.

[15] S. Latil and L. Henrard, Physical Review Letters 97(3), p. 036803, 2006.

[16] M. Aoki and H. Amawashi, Solid State Communications 142(3), pp. 123–127, 2007.

[17] P. Moon and M. Koshino, Physical Review B 87(20), p. 205404, 2013.

[18] S. Shallcross, S. Sharma, E. Kandelaki, and O. Pankratov, Physical Review B 81(16), p. 165105, 2010.

[19] T. Green and J. Weigle, "Theorie du moire," in Helvetica Physica Acta, 21(3-4), pp. 217–217, 1948.

[20] G. Trambly de Laissardiere, D. Mayou, and L. Magaud, Nano Letters 10(3), pp. 804–808, 2010.

[21] M. Rohlfing and S. G. Louie, Physical Review B 62(8), p. 4927, 2000.

[22] J. Ju, L. Wang, et al. Science, 358, 907910 (2017).

[23] H. Patel, R. W. Havener, L. Brown, Y. Liang, L. Yang, J. Park, and M. W. Graham, Nano Letters 15, 5932-5937, 2015.

[23] R. W. Havener, C.-J. Kim, L. Brown, J. W. Kevek, J. D. Sleppy, P. L. McEuen, and J. Park, Nano Letters 13(8), pp. 3942– 3946, 2013.

[24] Y. Zhang, T.-T. Tang, C. Girit, Z. Hao, M. C. Martin, A. Zettl, M. F. Crommie, Y. R. Shen, and F. Wang, " Nature 459(7248), pp. 820–823, 2009.

[25] R. W. Havener, Y. Liang, L. Brown, L. Yang, and J. Park, "Nano Letters 14(6), pp. 3353–3357, 2014.

[26] H. Patel, R. W. Havener, L. Brown, Y. Liang, L. Yang, J. Park, and M. W. Graham, Nano Letters 15(9), pp. 5932-5937, 2015.

[27] M.W. Graham, J. Chmeliov, Y.-Z. Ma, H. Shinohara, A.A. Green, M.C. Hersam, L. Valkunas, G.R. Fleming, *J Phys Chem B*, 5201–5211, 2011
